# Enamel Deproteinization with Sodium Hypochlorite: Effects on Ceramic Bracket Bond Strength

**DOI:** 10.1590/0103-644020256106

**Published:** 2025-04-14

**Authors:** Priscila Cordeiro, José Guilherme Neves, Midori Tsuzuki, Anália Gabriela Ferraz Facury, Lourenço Correr-Sobrinho, Ana Rosa Costa

**Affiliations:** 1 Department of Orthodontics, Hermínio Ometto Foundation - FHO, Araras, SP, Brasil.; 2 Department of Restorative Dentistry, Dental Materials area, Piracicaba Dental School, State University of Campinas, FOP - UNICAMP, Piracicaba, SP, Brazil; 3 Department of Restorative Dentistry, Universidade de Uberaba - UNIUBE, Uberaba, MG, Brazil

**Keywords:** Orthodontic brackets, Dental etching, Sodium hypochlorite, Shear Bond Strength, Scanning Electron Microscopy

## Abstract

To assess the efficiency of different concentrations of sodium hypochlorite (NaOCl) on the bonding of ceramic brackets to dental enamel using different bonding protocols. Ceramic, a polycrystalline bracket, was bonded to 90 extracted bovine incisors using primer + Transbond XT (P+TXT); Optibond S adhesive + Transbond XT (OS+TXT), and OS + Orthocem (OS+OC). Three concentrations of NaOCl (0%, 2.5%, and 5.25%) were used for each material, resulting in nine experimental groups (n=10). The shear bond strength (SBS) assay was performed on a universal testing machine. The surface morphology of the enamel and tooth/bonding material interface was examined under a scanning electron microscope. Kruskal-Wallis and Dunn’s tests were performed to compare the non-parametric data (ARI scores). SBS data were analyzed using a generalized linear model. All analyses were performed using the R program and with a significance level of 5%. No significant differences in SBS between the groups with different concentrations of NaOCl (p>0.05). OS+OC group yielded the lowest bond strength values (3.35 MPa_0%, 4.90 MPa_2.5%, 4.68 MPa_5.25%) compared to the other groups, regardless of NaOCl (p<0.05). No significant difference between the materials regarding the ARI score (p>0.05). OS+TXT-5.25% showed significantly higher scores (14.4 MPa) than OS+TXT-2.5% (9.17 MPa) (p<0.05). The surface morphology analysis revealed a change in the etching pattern for types 1 and 2 when NaOCl was used. The different concentrations of NaOCl previously applied onto the enamel surface did not enhance bond strength. OS+OC should not be recommended for bonding with fixed ceramic orthodontic devices.

## Resumo

Avaliar a eficiência de diferentes concentrações de hipoclorito de sódio (NaOCl) na colagem de braquetes cerâmicos ao esmalte dentário utilizando diferentes protocolos de colagem. Noventa incisivos bovinos foram colados com braquetes cerâmicos policristalino usando primer + Transbond XT (P+TXT); Optibond S adesivo + Transbond XT (OS+TXT) e OS + Orthocem (OS+OC). Três concentrações de NaOCl (0%, 2,5% e 5,25%) foram utilizadas para cada material, resultando em nove grupos experimentais (n=10). O ensaio de resistência de união ao cisalhamento (RUC) foi realizado em uma máquina de ensaio universal. A morfologia superficial do esmalte e da interface de união foi examinada no MEV. Os dados de RUC foram analisados por um modelo linear generalizado e os escores do IRA pelos testes de Kruskal-Wallis e Dunn no programa R, com nível de significância de 5%. Nenhuma diferença significativa foi observada na RUC entre os grupos com as diferentes concentrações de NaOCl (p>0,05). O grupo OS+OC apresentou os menores valores de resistência de união (3,35 MPa_0%, 4,90 MPa_2,5%, 4,68 MPa_5,25%) em relação aos demais grupos, independentemente do NaOCl (p<0,05). Não houve diferença significativa entre os materiais quanto ao escore do IRA (p>0,05). OS+TXT-5,25% apresentou escores (14,4 MPa) significativamente maiores do que OS+TXT-2,5% (9,17 MPa) (p<0,05). A análise da morfologia da superfície mostrou uma mudança no padrão de condicionamento para os tipos 1 e 2 quando o NaOCl foi usado. As diferentes concentrações de NaOCl, aplicadas previamente na superfície do esmalte, não aumentaram a resistência de união. OS+OC não deveria ser recomendado para colagem com braquetes ortodônticos cerâmicos.

**Figure:**
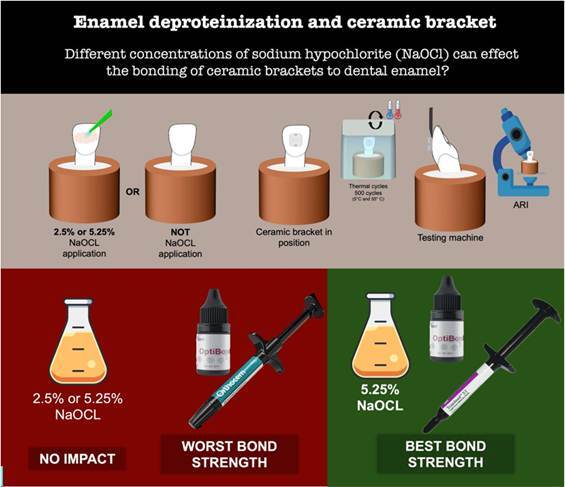

## Introduction

Failure at the bonding interface is clinically detected when frequent debonding of brackets occurs. Numerous techniques and materials have been developed in dentistry to enhance bond strength and improve the bonding of orthodontic devices to the dental enamel surface ^1^, such as glass ionomer bonding, self-etching primers, and powerful light-curing sources, along with self-ligating brackets and a different treatment concept: clear orthodontic aligners ^2^. These offer an alternative treatment approach by utilizing clear aligners instead of traditional brackets, thereby minimizing adhesive-related complications ^2^.

Three factors are necessary for successful bonding: the dental surface (size, contour, shape, and topography), the adhesive, and the bracket pad ^3^ ^,^ ^4^. In addition, other factors must be considered: remnants of the organic film and the plaque adsorbed and accumulated on the tooth surfaces. Therefore, the dental enamel surface should be prepared by removing any deposits or organic debris ^5^ and creating micropores or macropores for retention created through acid etching ^1^. Tooth enamel, primarily composed of hydroxyapatite, contains a low amount of proteins that affect its interaction with adhesives used in orthodontics. This mineral-rich structure necessitates effective surface treatment to create micropores that enhance mechanical retention, ultimately improving bond strength between the enamel and orthodontic devices ^1^.

In dentistry, phosphoric acid at 37%, with an etching time ranging from 15 to 30 seconds, is commonly used for the preparation of dental enamel, activated by friction and rinsing throughout the etching procedure ^6^ ^,^ ^7^ ^,^ ^8^. The action of phosphoric acid on the dental enamel surface occurs primarily on the mineralized or inorganic component (96%). Organic matter (1%) is not eliminated, and its presence can hinder or prevent the improvement of the etching pattern ^9^.

Several techniques can be employed before dental enamel etching to obtain an adhesive interface with better physical and mechanical properties. Such techniques include conventional cleaning with pumice and a rubber cup, air polishing systems, sonication, lasers, and deproteinizing agents ^1^ ^,^ ^6^ ^,^ ^7^ ^,^ ^8^ ^,^ ^10^ ^,^ ^11^ ^,^ ^12^. In daily dental clinical practice, deproteinization can be carried out quickly; it is a convenient, non-invasive, and inexpensive method for removing organic material from dental enamel before etching, thus enhancing the bond strength of restorative materials and orthodontic devices ^8^.

Currently, different deproteinizing agents have been utilized, such as Papacárie® ^13^; papain gel (10%) ^7^ ^,^ ^8^ ^,^ ^13^; bromelain, an enzyme found in the leaves of the pineapple plant ^14^; hydrogen peroxide (3.5%) ^15^; sodium hypochlorite (NaOCl) at 2.5% ^10^; and NaOCl at 5.25% ^1^ ^,^ ^6^ ^,^ ^7^ ^,^ ^8^ ^,^ ^9^ ^,^ ^2^ ^,^ ^16^ ^,^ ^17^ ^,^ ^18^ ^,^ ^19^ ^,^ ^20^ ^,^ ^21^. The use of NaOCl for this purpose seems to be a viable and effective choice, as it possesses good antimicrobial activity, high cleaning power, and biocompatibility, without damaging healthy tissue or tooth structure ^6^ ^,^ ^7^ ^,^ ^8^ ^,^ ^12^. These properties relate to chemical reactions such as saponification, amino acid neutralization, and chloramination ^22^. In dental enamel, NaOCl penetrates the rods after the removal of the organic film formed prior to etching, allowing the etchant to efficiently infiltrate the dental enamel, creating type 1 and type 2 etching patterns ^1^ ^,^ ^6^ ^,^ ^7^ ^,^ ^8^ ^,^ ^11^ ^,^ ^12^ ^,^ ^16^ ^,^ ^17^ ^,^ ^18^.

The selection of the NaOCl concentration is based directly on the desired effect and on the principle of preventing any damage to the dental tissues ^23^. As for deproteinization, no consensus exists on which NaOCl concentration would be ideal. Note that most studies on deproteinization with NaOCl have used metal brackets and a concentration of 5.25% ^1^ ^,^ ^6^ ^,^ ^7^ ^,^ ^8^ ^,^ ^9^ ^,^ ^11^ ^,^ ^12^ ^,^ ^16^ ^,^ ^17^ ^,^ ^18^ ^,^ ^19^ ^,^ ^20^ ^,^ ^21^, except for Abdelmegid ^10^, who employed NaOCl at 2.5%. Moreover, a recent study ^12^ assessed the action of NaOCl at 5.25% on monocrystalline ceramic brackets.

Ceramic brackets have been used to meet patients’ aesthetic needs. Since their introduction into the market, efforts have been made to improve the bonding of brackets to the teeth by treating and/or including retainers in the bracket pad and deproteinization of the dental enamel surface ^12^. Although several studies have explored enamel deproteinization using NaOCl, comparisons between different concentrations of NaOCl (2.5% and 5.25%) combined with monocrystalline ceramic brackets and various bonding materials are still lacking. Therefore, this study aims to fill this gap in the literature by evaluating the effects of these concentrations on bond strength.

Accordingly, the present study aimed to assess the efficiency of NaOCl at 2.5% and 5.25% in improving the bond strength of ceramic brackets to the dental enamel using different combinations of bonding materials. The following null hypotheses were stated: 1) The different combinations of bonding materials would not affect the bond strength, and 2) NaOCl at different concentrations would not interfere with the mechanical properties of the materials.

## Materials and methods

### Selection and preparation of teeth and solutions

A total of 135 extracted bovine incisors were used, following these criteria: intact dental enamel surface, free of cracks, not previously treated with chemical agents, and free of developmental defects or lesions or hypomineralized caries. Immediately after extraction, the extracted teeth were thoroughly washed in running water and cleaned in 0.1% thymol solution at room temperature. The teeth were stored in distilled water in a refrigerator (4°C) for up to a week before starting the experiment.

A Ceramic® polycrystalline bracket, Roth prescription, 0.022” x 0.028” slot size, and 14.00 mm^2^ area was utilized (Morelli, Sorocaba, SP, Brazil). NaOCl solutions (2.5% and 5.25%) were manipulated at a compounding pharmacy (FHO, Araras, SP, Brazil).

### Experimental groups

**Figure 1:**
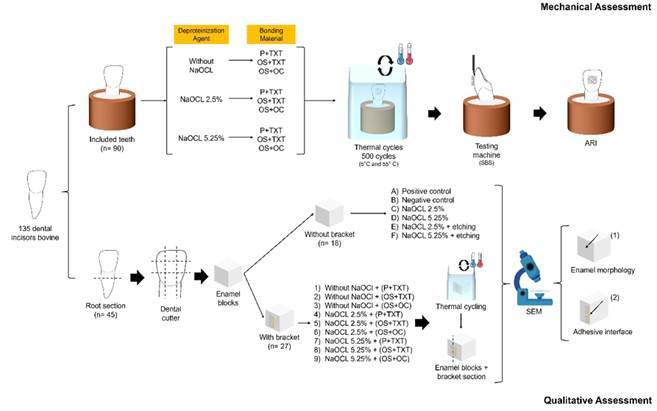
Experimental Design: Mechanical and Qualitative Evaluation. *Note: (P) Transbond XT Primer (3M Unitek); (TXT) Transbond XT Light Cure Adhesive (3M Unitek); (OS) OptiBond S (Kerr); (OC) Orthocem (FGM); (NaOCl) sodium hypochlorite; (SBS) shear bond strength; (ARI) adhesive remnant index; (SEM) scanning electron microscopy.

The present study was conducted according to the experimental design illustrated in Figure 1. For mechanical evaluation, the teeth were embedded in a polyvinyl chloride (PVC) cylinder containing chemically activated acrylic resin (JET, Campo Limpo Paulista, SP, Brazil), with the buccal surface perpendicular to the horizontal axis, and randomly organized into nine groups (n=10) according to the type of bonding material used for bracket placement. The sample size of 10 specimens per group was predetermined based on previous literature, specifically following the methodology outlined by Mahmoud et al. ^6^. This study assumed a significance level of 0.05 and a study power of 80% for evaluating adhesive bonding with enamel deproteinization, providing a reliable foundation for our sample size selection.

**Figure 2:**
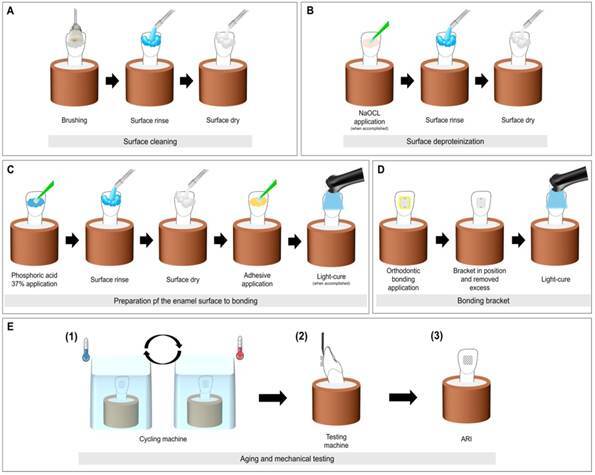
Preparation of teeth for mechanical assessment. (A) Surface cleaning. (B) Surface deproteinization, when accomplished. (C) Preparation of the enamel surface for bonding. (D) Bonding brackets. (E) Aging and mechanical testing.

Before bonding, the buccal surfaces of the bovine teeth were cleaned and polished with pumice paste (S.S. White, Rio de Janeiro, RJ, Brazil) and deionized water, using a Robson brush (Microdont, São Paulo, SP, Brazil) mounted on a contra-angle handpiece operated at low speed (Dabi Atlante, SP, Brazil) for 15 s. The brush was replaced every ten prophylactic procedures. Subsequently, the teeth were rinsed under running water for 10 s and dried with air jets (Figure 2 - A). 

NaOCl (2.5% and 5.25%) (Figure 2 - B), phosphoric acid at 37%, adhesive (Figure 2 - C), and orthodontic bonding materials for bracket placement were applied according to the manufacturer’s instructions (Table 1).

Before applying the orthodontic bonding material on the bracket pad, a thin layer of the adhesives (Transbond XT primer (P) - 3M Unitek, Monrovia, California, USA - or Optibond S adhesive system (OS) - Kerr Corporation Orange, California, USA) was applied, covering the whole retention area, before the application of the Transbond XT Light Cure Adhesive (TXT) (3M Unitek, Monrovia, California, USA) orthodontic bonding or Orthocem (OC) (FGM, Joinville, SC, Brazil) orthodontic bonding, respectively, as recommended by the manufacturer.

Thereafter, the bonding material was applied to the bracket pad and firmly pressed onto the dental enamel surface. Excess resin around the bracket pad was removed with an exploratory probe. The materials for the bonding of orthodontic accessories (TXT and OC) were light-cured for 10 s on each surface (total of 40 s), using an LED Valo device (Ultradent Products Inc., South Jordan, UT, USA) at an irradiance of 1,000 mW/cm^2^, which had been previously measured with a radiometer (Model 100, Demetron Research Corporation, Danbury, CT) (Figure 2 - D).

After polymerization, the set was kept in deionized water at 37°C for 24 h in an oven. The specimens were then subjected to 500 thermal cycles with deionized water on a cycling machine (MSCT 3; Marnucci ME, São Carlos, SP, Brazil) between 5º and 55ºC (with an immersion time of 30 s at each temperature) and 6 s of transfer between baths ^6^ ^,^ ^25^ (Figure 2 - E ^1^).

**Table 1:** Composition, manufacturer, and mode of application of materials used in the present study.

Products	Composition (% weight)	Manufacturer	Mode of application
Condac (etcher enamel)	Phosphoric acid at 37%.	FGM, Joinville, SC, Brazil	Apply phosphoric acid at 37% for 15 s on the enamel; rinse for 15 s (water + spray) and dry with oil-free compressed air jet for 5 s.
NaOCl (deproteinization agente)	2.5 and 5.25%.	FHO, Araras, São Paulo, Brazil	Apply NaOCl actively for 60 s on the enamel using a microbrush, rinse, and dry for 10 s.
Optibond S (adhesive)	Ethyl alcohol (20-25), dimethacrylates (55-60), barium aluminosilicate glass (5-10), silicon dioxide (5-10), sodium hexafluorosilicate (0.5-1).	Kerr Corporation Orange, California, USA	Slightly apply a thin layer of adhesive system for 15 s on the enamel using a microbrush, dry with light air jet for 3 s, and light-cure for 20 s.
Transbond XT Primer (adhesive)	Bis-GMA (45-55), TEGDMA (45-55), triphenylamine (<1), CQ (<0.3), DMAEMA (<0.5), hydroquinone (<0.03).	3M Unitek, Monrovia, California, USA	Actively apply the primer for 10 s on the enamel using a microbrush and dry with light air jet for 5 s.
Transbond XT Light Cure Adhesive (orthodontic bonding)	Bis-GMA (10-20), BIS-EMA (5-10), silanized quartz (70-80), silanized silica (<2), diphenyl iodonium hexafluorophosphate (0.2).	3M Unitek, Monrovia, California, USA	Apply the resin on the bracket pad and light-cure it for 10 s on each surface.
Orthocem (orthodontic bonding)	Bis-GMA, TEGDMA, phosphate methacrylate monomers, CQ, tertiary amine, silicon dioxide.	FGM, Joinville, SC, Brazil	Apply the resin on the bracket pad and light-cure it for 10s on each surface.

### Shear bond strength (SBS)

After thermal cycling, the specimens were subjected to the SBS assay on a universal testing machine (Instron, Model 4411, Canton, MA, USA). The applied force was parallel to the tooth surface at the top of each bracket pad using a chisel at a 1.0 mm/min speed and a 50 N load cell until fracture occurred (Figure 2 - E ^2^). The force at debonding was recorded in Newton and converted to MPa by applying the following formula ^7^:

$${MP}_{a}=\frac{Force(N)}{Bracket\;surface\;area\;({mm}^{2})}$$

Roth Ceramic area (Morelli) = 14.00 mm^2^. The area was calculated by multiplying the width and height of the bracket pad (3.5 mm x 4.0 mm).

### Adhesive remnant index (ARI)

After debonding, the tooth and bracket surfaces were examined under a stereoscopic microscope (Olympus Corp, Tokyo, Japan) at a magnification of 25x. ARI was used to classify the failure modes according to Årtun and Bergland ^24^: 0 - no resin adhered on the dental enamel; 1 - less than 50% of the resin adhered on the enamel; 2 - more than 50% of the resin adhered on the enamel; and 3 - all of the resin was adhered on the enamel, with a distinct impression of the bracket mesh (Figure 2 - E ^3^).

### Scanning electron microscopy (SEM)

For qualitative assessment (SEM), after prophylaxis, 45 healthy bovine teeth (18 for enamel morphology and 27 for adhesive interface) had their roots sectioned 1 mm beyond the cementoenamel junction using a diamond disc (Extec Corp., CT, USA) mounted on a contra-angle handpiece operated at low speed (Dabi Atlante, SP, Brazil). After that, the crowns were cut perpendicularly to the long axis of the tooth, in the mesiodistal direction, with a double-sided disc (Extec Corp., CT, USA) under water cooling on a cutting machine (Isomet, Buehler Ltd., Lake Buff, IL, USA), resulting in blocks obtained from the buccal surface (5 x 5 x 5 mm), with one block per tooth (Figure 1 - Qualitative Assessment).

### Enamel surface morphology

The dental enamel blocks were randomly assigned to six groups (n=3) for topographic assessment of the dental enamel: A - healthy enamel surface (positive control); B - healthy enamel surface etched with H_3_PO_4_ at 37% for 15 s (negative control); C - healthy enamel surface after application of the deproteinizing agent (NaOCl at 2.5%) for 60 s; D - healthy enamel surface after application of the deproteinizing agent (NaOCl at 5.25%) for 60 s; E - healthy enamel surface after application of NaOCl 2.5% for 60 s + etching with H_3_PO_4_ at 37% for 15 s; and F - healthy enamel surface after application of NaOCl 5.25% for 60 s + etching with H_3_PO_4_ at 37% for 15 s (Figure 1 - Qualitative Assessment ^1^).

The specimens were coated with gold/palladium (Balzers-SCD 050 sputter coater, Germany). Two different locations were randomly selected for microscopic analysis on each specimen. The images of each location were captured at a magnification of 1,000 x using SEM (JEOL-5600 LV, Japan) at an accelerating voltage of 20 KV and working distance (WD) of 9.2 to 10.9 mm.

The images were evaluated according to the etching patterns and topographic characteristics of the dental enamel and compared with three basic etching patterns: type 1, type 2, and type 3. Types 1 and 2 are the ideal etching patterns for obtaining an appropriate bond strength ^5^.

### Adhesive interface (tooth/bonding material)

The dental enamel blocks were randomly assigned to nine groups (n=3) for the assessment of adhesive interface, based on the type of bonding material (P+TXT; OS+TXT; OS+OC) and on the presence or absence of a previous deproteinizing agent - without NaOCl (control) and with NaOCl (2.5% and 5.25%). As previously described, the tooth/bracket set was subjected to thermal cycling.

The blocks with the positioned brackets were sectioned in half in the buccopalatal direction using a diamond disc (Extec Corp., CT, USA) on a metallographic cutting machine (Isomet 4000, Buehler, USA) under water cooling and at low speed (Figure 1 - Qualitative Assessment ^2^). Subsequently, the surfaces were cleaned with distilled water and sonicated for 30 min.

The specimens were mounted on metal stubs and coated with gold/palladium (Balzers-SCD 050 sputter coater, Germany). Two different locations were randomly chosen on each specimen for microscopic analysis. The images of each location were captured at a magnification of 1000 x using SEM (JEOL-5600 LV, Japan), under an accelerating voltage of 20 KV and working distance (WD) of 33.1 to 33.7 mm.

### Statistical analysis

Descriptive statistics, including means and standard deviations, were initially computed to summarize the SBS and ARI data. Exploratory data analysis, such as visual inspection using boxplots and histograms, was performed to assess the distribution and detect potential outliers. These analyses indicated that the data do not meet the assumptions of classical analysis of variance with a general linear model. Consequently, the SBS data were analyzed using a generalized linear model, considering the effects of material, NaOCl concentration, and their interaction. Non-parametric tests of Kruskal-Wallis and Dunn were carried out for ARI scores. The statistical analyses were performed using the R program, version 4.4.1 (R Core Team, 1999-2024, Vienna, Austria), with a significance level of 5%.

## Results

### SBS (MPa)

SBS values are shown in Table 2. The results indicate that there was no significant difference between the groups with different concentrations of sodium hypochlorite in terms of shear bond strength (table 2). Regardless of NaOCl concentration, the OS+OC group had significantly lower SBS than the other two groups (p<0.05).

**Table 2:** Shear bond strength (MPa) of ceramic brackets bonded onto the bovine dental surface with or without hypochlorite sodium (NaOCl) application, using two different resins and adhesive systems.

	NaOCl concentration					
	0% (without)		2.5%		5.25%	
	Mean (Sd)	Median (Min; Max value)	Mean (Sd)	Median (Min; Max value)	Mean (Sd)	Median (Min; Max value)
P+TXT	14.02 (6.23) Aa	13.55 (5.80; 26.20)	10.42 (4.74) Aa	8.65 (6.40; 22.90)	10.41 (5.04) Aa	8.80 (6.10; 22.10)
OS+TXT	11.90 (8.55) Aa	7.50 (3.20; 28.40)	9.17 (5.25) Aa	7.70 (2.80; 18.50)	14.40 (8.12) Aa	15.50 (2.60; 26.90)
OS+OC	3.35 (1.76) Ab	4.00 (1.00; 5.50)	4.90 (3.43) Ab	3.95 (1.60; 12.30)	4.68 (1.09) Ab	4.75 (1.90; 8.20)

p(material)<0.0001; p(concentration)=0.5693; p(interaction)=0.2046. Distinct letters (uppercase horizontally and lowercase vertically) indicate statistically significant differences (p≤0.05).

* Note: (P) Transbond XT primer (3M Unitek); (TXT) Transbond XT resin (3M Unitek); (OS) - OptiBond S adhesive system (Kerr); (OC) - Orthocem resin (FGM); (NaOCl) - Sodium hypochlorite. (Sd) Standard deviation. (Min) Minimum. (Max) Maximum.

### ARI

ARI values are shown in Table 3. There was no significant difference between the materials regarding the ARI score (p>0.05), as shown in Table 3. In the OS+TXT group, the score was significantly higher at the 5.25% NaOCl concentration than at the 2.5% concentration (p<0.05).

**Table 3:** Adhesive remnant index (ARI) score as a function of bonding material and NaOCl concentration.

	Deproteinizing agent (NaOCl)															
	0% (without)					2.5%					5.25%					
	ARI score					ARI score					ARI score					
	0	1	2	3		0	1	2	3		0	1	2	3		*p-value*
P+TXT	2	1	1	6	Aa	3	1	2	4	Aa	0	2	2	6	Aa	0.4741
OS+TXT	0	2	3	5	Aba	2	4	3	1	Ba	0	0	3	7 (1CBF)	Aa	0.0067
OS+OC	0	5	4	1	Aa	0	5	4	1	Aa	0	3	5	2	Aa	0.5420
*p-value*	0.2301					0.6521					0.0766					

Distinct letters (uppercase horizontally and lowercase vertically) indicate statistically significant differences (p≤0.05). (P) Transbond XT primer (3M Unitek); (TXT) Transbond XT resin (3M Unitek); (OS) - OptiBond S adhesive system (Kerr); (OC) - Orthocem resin (FGM); (NaOCl) - Sodium hypochlorite. ARI scores: 0, no adhesive left on enamel surface; 1, less than half of adhesive left; 2, more than half of adhesive left; and 3, all adhesive left with a distinct imprint of the bracket mesh. CBF - Ceramic bracket failure.

### Surface Morphology (SEM)

**Figure 3:**
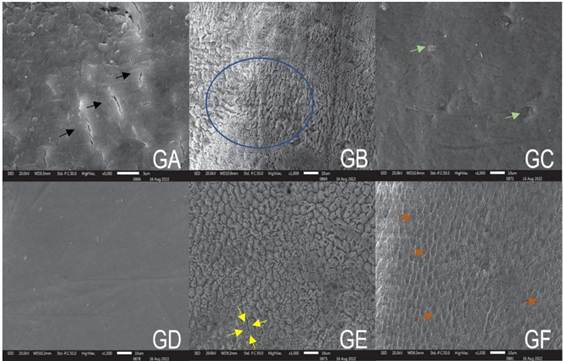
SEM images of enamel surface treatment using different techniques, 1000x magnification: GA. Healthy enamel (positive control); GB. H_3_PO_4_ at 37% (negative control); GC. NaOCl 2.5%; GD. NaOCl 5.25%; GE. NaOCl 2.5% + H_3_PO_4_ 37%; and GF. NaOCl 5.25% + H_3_PO_4_ 37%. GA. Irregular surface with defects and organic debris (black arrows). GB. Random pattern showing etching types 1 and 2 (type 3; blue circle). GC. Less irregular surface with small pits and debris (green arrows). GD. Regular surface without debris. GE. The periphery of enamel rods was removed, with intact nuclei (type 2; yellow arrows). GF. The periphery of intact enamel rods and nuclei was removed (type 1; orange arrows).

The results for the surface morphology of the enamel after different treatments are shown in Figure 3.

### Adhesive Interface (SEM)

**Figure 4:**
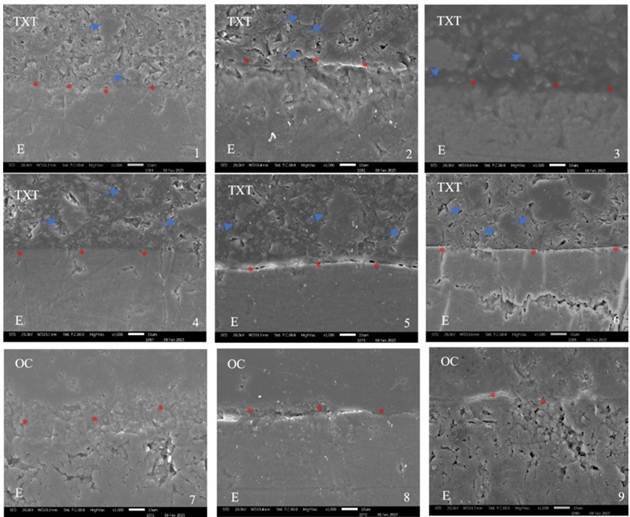
SEM images of the adhesive interface (base of the ceramic orthodontic accessory/bovine enamel) for the different groups at 1000x magnification. Images 1, 2, and 3: P+TXT-0%, 2.5%, and 5.25%, respectively; Images 4, 5, and 6: OS+TXT-0%, 2.5%, and 5.25%, respectively; and Images 7, 8, and 9: OS+OC-0%, 2.5%, and 5.25%, respectively. TXT - Transbond XT Light Cure Adhesive (orthodontic bonding). OC - Orthocem (orthodontic bonding). E - Bovine enamel. Presence of inorganic loads in Transbond XT Light Cure Adhesive (blue arrows). Asterisks in orange indicate a continuous adhesive interface (Figs. 1-4 and 6) and an irregular interface with defects (Figs. 5, 7, 8, 9).

The adhesive interface (orthodontic accessory/bovine enamel) after different bonding protocols (P+TXT, OS+TXT, OS+OC), either associated or not with the application of NaOCl, is shown in Figure 4.

## Discussion

The first null hypothesis was rejected because the different combinations of bonding materials interfered with bond strength. The groups that used primer + Transbond XT (P+TXT) and OptiBond S + Transbond XT (OS+TXT) showed satisfactory bond strength. According to a review by Cruz et al. ^11^, values between 6 and 8 MPa are clinically acceptable and able to withstand forces during orthodontic treatment. Replacement of the primer with an adhesion-promoting agent previously applied to the Transbond XT bonding resin, albeit not statistically significant (Table 2), caused a reduction in SBS in the group without and with deproteinization with NaOCl at 2.5%. The primer, thanks to its flowability (lower viscosity), penetrates the micropores created by acid etching, thus increasing mechanical interlocking between the enamel and the resin ^18^. The findings of the present study, however, are at odds with those obtained by Sharma et al. ^8^, who observed the use of a bonding agent with Transbond XT significantly increased bond strength. Therefore, the difference in the results across the studies might have occurred due to the difference in their methodology involving, for instance, the substrate, adhesive system, and orthodontic accessory.

In this study, the OS+OC 0%, 2.5%, and 5.25% groups (Table 2) yielded clinically unacceptable SBS; therefore, these materials are not recommended for the placement of polycrystalline ceramic brackets. Previous studies have not corroborated the findings of the present study in that the Orthocem resin either associated or not with an adhesive system met the clinical requirements ^25^ ^,^ ^26^ ^,^ ^27^. It should be underscored, however, that the referenced studies used metal brackets and that the difference in SBS is probably due to the type and textured pattern of bracket pads ^25^ ^,^ ^26^ ^,^ ^27^.

NaOCl at different concentrations did not interfere with the mechanical properties of the materials. Consequently, the second null hypothesis was accepted. The application of NaOCl did not affect SBS, regardless of the bonding material used (Table 2). These findings are in line with those of a previous study ^19^, but it is important to highlight that SBS was determined for resin Transbond XT and metal bracket. Conversely, other studies have shown that deproteinization with NaOCl at 5.25% interfered with SBS on account of the bonding material used ^6^ ^,^ ^18^. In such studies, bonding material that does not utilize a primer or adhesive system plays a key role in the prior use of NaOCl before acid etching, promoting a better etching pattern of type 1 or 2.

 Thus, as with the studies conducted by Justus et al. ^18^ and Mahmoud et al. ^6^, no statistical difference was observed in the P+TXT-0%, 2.5%, and 5.25% groups. Primer + Transbond XT resin is still the best option for the bonding of brackets because of its appropriate mechanical properties and bonding technique, which reduce the number of steps, chair time, and contamination during the bonding procedures, as also described in previous studies ^25^ ^,^ ^26^ ^,^ ^27^. Notably, the SBS of OS+TXT-5.25% was like that of P+TXT-0%, regarded as the gold standard in dentistry. Despite showing no statistically significant difference, the application of NaOCl at 5.25% before the use of OS+TXT improved cleaning and/or removal of organic matter, ameliorating type 1 and 2 etching patterns, in line with SEM findings for surface morphology (Fig. 2). Interestingly, there was no statistically significant difference between OS+TXT-5.25% and the other groups (OS+TXT-2.5% and 0%).

Regarding ARI, score 3 was predominant in the groups P+TXT and OS+TXT, except OS+TXT-2.5%NaOCl (Table 3). Although there was no significant difference between the materials regarding the ARI score (Table 3), higher ARI can be attributed to the greater adhesion of the resin to the enamel surface, indicating an accessory adhesive failure ^7^. These findings are at odds with those previous studies ^6^ ^,^ ^8^ ^,^ ^18^ ^,^ ^20^ in which experimental groups subjected to deproteinization with NaOCl had higher scores, with a larger amount of residual resin in the dental enamel, except in the OS+TXT-5.25% that had a significantly higher score than OS+TXT-2.5% (Table 3). As for the OS+OC group, deproteinization with NaOCl-5.25% changed the score, which was 1 in the untreated group and NaOCl-2.5% group, to 2, thus indicating an accessory adhesive failure, in line with the results obtained in the present study for the adhesive interface (Fig. 3). These two types of failures have advantages and disadvantages. An accessory adhesive failure indicates good adhesion onto the enamel, but it requires a longer chair time to remove the residual adhesive, posing a high risk of damage to the enamel, depending on the technique used ^7^ ^,^ ^8^ ^,^ ^28^. On the other hand, in the enamel adhesive failure, less adhesive remains on the enamel, thus making cleaning easier, but there is a risk of microfracture of the enamel during debonding of the accessory; in addition, debonding probably occurs more frequently during the treatment, increasing chair time and orthodontic treatment length ^25^ ^,^ ^26^ ^,^ ^27^.

The main factors for bonding failure are related to the amount of etched surface and the quality of the etching pattern ^7^. In addition to the mechanical aspects of adhesion, it is essential to consider the clinical implications of our findings for orthodontic practice. The improvement in bond strength of ceramic brackets through enamel deproteinization with NaOCl may result in a significant reduction in debonding rates during orthodontic treatment. This reduction not only enhances treatment efficiency but also minimizes patient discomfort and consultation time, essential factors for patient compliance with treatment. Therefore, the results of this study provide a foundation for optimizing bonding techniques, potentially elevating the quality of orthodontic care and clinical outcomes.

In the present study, after the respective enamel surface treatments in each group (Fig. 2), GA (enamel prophylaxis) and GC (NaOCl at 2.5%) revealed a sizable amount of organic debris on the enamel surface, whereas GD (NaOCl at 5.25%) showed less debris. The group etched with H_3_PO_4_ at 37% (GB) predominantly exhibited a type 3 etching pattern. Conversely, the specimens subjected to deproteinization with NaOCl at 2.5% and 5.25% before acid etching (H_3_PO_4_ at 37%) (GE and GF) revealed a predominance of type 2 and 1 pattern, respectively, which are better for orthodontic bonding, given that the porous surface provides larger and deeper retention ^27^. Similar findings were observed by López-Luján et al. ^1^, Panchal et al. ^7^, Al-Daher et al. ^16^, and Justus et al. ^18^. Nevertheless, Ahuja et al. ^9^ claimed that H_3_PO_4_ had the best etching pattern, thus suggesting it as the best therapeutic option. The control groups (no application of NaOCl) exhibited a type 3 pattern after phosphoric acid etching and were not statistically different from the groups to which NaOCl was applied prior to etching, which exhibited type 1 and 2 patterns. Hence, the application of the primer and/or adhesive system is seemingly more important than the type of etching pattern on the enamel surface. The primer and/or adhesive system penetrates the micropores created by the etching and, consequently, forms a microscopic mechanical interlocking between the enamel and the resin ^18^.

Note that all specimens were subjected to thermal cycling after bracket placement, following the recommendations of previous studies on NaOCl application and metal brackets ^6^ ^,^ ^18^. In orthodontics, the number of thermal cycles varies from 500 to 6,000, corresponding to 0.42 (approximately 1 year) and 5 years, respectively ^29^. During thermal cycling, the specimens undergo thermal changes, producing shrinkage/expansion and additional exposure to water and/or solubilization of the bonding materials, thereby affecting the adhesion of the resin onto the bracket and the tooth, possibly leading to bonding failure ^29^. The different protocols used in the studies do not allow standardizing the in vitro measurements of SBS, the media for preservation of the tooth, the type of tooth, and the adhesive materials, among others ^20^. This hinders the comparison of the findings of the present study with those of other in vitro studies.

Future research should focus on increasing the number of thermal cycles, storage time, and including other bonding materials, adhesive systems, and human teeth. Moreover, the treatment of the ceramic bracket pad with hydrofluoric acid etching, followed by silanization, could be performed prior to the placement of the bonding material instead of applying the primer or adhesive system as done in the present study.

## Conclusion

This study demonstrated that the use of sodium hypochlorite as a deproteinizing agent did not affect shear bond strength, regardless of the bonding material employed. Additionally, the combination of OptiBond S and Orthocem, both with and without sodium hypochlorite application, exhibited significantly lower bond strength compared to other materials. Therefore, due to their inadequate bond strength, OptiBond S and Orthocem are not recommended for use in bonding polycrystalline ceramic brackets.
